# What if: A retrospective reconstruction of resection cavity stereotactic radiosurgery to mimic neoadjuvant stereotactic radiosurgery

**DOI:** 10.3389/fonc.2023.1056330

**Published:** 2023-03-16

**Authors:** Gueliz Acker, Marcel Nachbar, Nina Soffried, Bohdan Bodnar, Anastasia Janas, Kiril Krantchev, Goda Kalinauskaite, Anne Kluge, David Shultz, Alfredo Conti, David Kaul, Daniel Zips, Peter Vajkoczy, Carolin Senger

**Affiliations:** ^1^ Department of Neurosurgery, Charité-Universitätsmedizin Berlin (Corporate Member of Freie Universität Berlin, Humboldt-Universität zu Berlin, and Berlin Institute of Health), Berlin, Germany; ^2^ Berlin Institute of Health at Charité - Universitätsmedizin Berlin, BIH Academy, Clinician Scientist Program, Berlin, Germany; ^3^ Department of Radiation Oncology and Radiotherapy, Charité-Universitätsmedizin Berlin (Corporate Member of Freie Universität Berlin, Humboldt-Universität zu Berlin, and Berlin Institute of Health), Berlin, Germany; ^4^ Department of Radiation Oncology, University of Toronto, Toronto, ON, Canada; ^5^ Department of Biomedical and Neuromotor Sciences, Alma Mater Studiorum - Università di Bologna, Bologna, Italy; ^6^ German Cancer Consortium (DKTK), Partner Site Berlin, German Cancer Research Center (DKFZ), Heidelberg, Germany

**Keywords:** neoadjuvant, stereotactic radiosurgery (SRS), CyberKnife^®^, brain metastases (BM), preoperative

## Abstract

**Introduction:**

Neoadjuvant stereotactic radiosurgery (NaSRS) of brain metastases has gained importance, but it is not routinely performed. While awaiting the results of prospective studies, we aimed to analyze the changes in the volume of brain metastases irradiated pre- and postoperatively and the resulting dosimetric effects on normal brain tissue (NBT).

**Methods:**

We identified patients treated with SRS at our institution to compare hypothetical preoperative gross tumor and planning target volumes (pre-GTV and pre-PTV) with original postoperative resection cavity volumes (post-GTV and post-PTV) as well as with a standardized-hypothetical PTV with 2.0 mm margin. We used Pearson correlation to assess the association between the GTV and PTV changes with the pre-GTV. A multiple linear regression analysis was established to predict the GTV change. Hypothetical planning for the selected cases was created to assess the volume effect on the NBT exposure. We performed a literature review on NaSRS and searched for ongoing prospective trials.

**Results:**

We included 30 patients in the analysis. The pre-/post-GTV and pre-/post-PTV did not differ significantly. We observed a negative correlation between pre-GTV and GTV-change, which was also a predictor of volume change in the regression analysis, in terms of a larger volume change for a smaller pre-GTV. In total, 62.5% of cases with an enlargement greater than 5.0 cm^3^ were smaller tumors (pre-GTV < 15.0 cm^3^), whereas larger tumors greater than 25.0 cm^3^ showed only a decrease in post-GTV. Hypothetical planning for the selected cases to evaluate the volume effect resulted in a median NBT exposure of only 67.6% (range: 33.2–84.5%) relative to the dose received by the NBT in the postoperative SRS setting. Nine published studies and twenty ongoing studies are listed as an overview.

**Conclusion:**

Patients with smaller brain metastases may have a higher risk of volume increase when irradiated postoperatively. Target volume delineation is of great importance because the PTV directly affects the exposure of NBT, but it is a challenge when contouring resection cavities. Further studies should identify patients at risk of relevant volume increase to be preferably treated with NaSRS in routine practice. Ongoing clinical trials will evaluate additional benefits of NaSRS.

## 1 Introduction

The incidence of brain metastases in patients with solid tumors is estimated to be as high as 20.0% to 30.0% and is increasing due to improvements in systemic treatments and diagnostic imaging (1, 2). Consequently, and due to better control of the primary tumor and extracranial metastases, the treatment of brain metastases is gaining importance. Surgical resection of large or symptomatic tumors is most often the first treatment step followed by irradiation, as several randomized trials have demonstrated better local control with postoperative whole brain radiation therapy (post-WBRT) or postoperative stereotactic radiosurgery (post-SRS) compared to surgery alone (3–5). With increasing life expectancy due to individualized treatment approaches, there is a need to prevent cognitive impairment, which is why SRS is coming to the fore as a replacement for WBRT. Subsequently, Brown et al. compared post-WBRT with post-SRS in a randomized phase III study showing a better cognition-deterioration-free survival in post-SRS cohort with comparable overall survival in both groups. However, local control rates were worse after post-SRS (6). However, this could be due to the wide dose range, which in this study design was as low as 12 Gy in a single fraction, reflecting the need for improvement in this treatment regimen. In this context, El Safie et al. performed a comparison that also included hypofractionated SRS (HF-SRS) and found a 12-month local control rate of 94.9% for SRS/HF-SRS versus 81.7% for WBRT (7). A previous study from Patel et al. also presented 1-year local control (LC) 83% for SRS including single and HF-SRS vs. 74% for WBRT (8). Kepka et al. performed a randomized trial on this, but failed to demonstrate the non-inferiority of SRS to WBRT in terms of local control most likely due to underpowering (9). Taken together, further randomized trials are warranted including HF-SRS instead of using too low single doses.

In addition to the dose scheme issue that has to be further optimized, one further important limitation of post-SRS is probably the uncertainty of target delineation, as evidenced by the wide range of contours for ill-defined resection cavities in the contouring guidelines (10). This was also reflected in the comparative simulation study of Vellayappan et al. with high interobserver variability in resection cavity target delineation (11). Therefore, the usual practice to expand the margins, in addition to the resection cavity and to cover the surgical tracts and meninges along the bone flap, may result in larger volumes than the metastases themselves, exposing more normal brain tissue (NBT) to radiation (10, 12). Another potential pitfall of post-SRS is leptomeningeal disease (LMD), which has been reported to account for up to 35% (13, 14). Reported risk factors for LMD include primary tumor entities of the intracranial metastasis (15–18), number of intracranial lesions (13, 16, 17, 19), prior resection of an intracranial lesion (18–20), no additional immunotherapy (20, 21) and hemorrhagic or cystic features of the lesion (17), although the results of univariate and multivariate analysis vary amongst these papers. Importantly, Foreman et al. reported that they found no significant differences in LMD rates after SRS and HF-SRS (13). If we look at the above-mentioned comparative studies, Patel et al. reported WBRT to be associated with a significantly lower rate of LMD occurrence compared to SRS alone (18-month LMD 13% vs. 31%, log-rank P = 0.045); however, they did not assess the influence of SRS and HF-SRS (8).

In view of the disadvantages presented and with the aim of improving local control, neoadjuvant radiosurgical treatment (NaSRS) of intact brain metastases is currently attracting increasing attention. To date, there are nine published studies that have evaluated the efficacy of NaSRS with encouraging results, however, they are mainly retrospective in design (**Table 1**) (19, 22–27, 29, 30). Although a possible reduction in target volume and better sparing of healthy brain tissue have been frequently proposed as potential advantages of NaSRS, no detailed analysis of these benefits has been published. For instance, Udovicich et al. demonstrated a larger target volume after resection in a representative case (29), whereas Vellayappan et al. could not confirm a smaller volume preoperatively in a small cohort of ten patients after simulation of a NaSRS treatment (11). Atalar et al. reported that resection cavities were smaller than the target volume before resection in most cases, but without considering the recommended margin of 2 to 3 mm for planning target volume (PTV) (31, 32). In this regard, a very recent study by Bugarini et al. observed a tendency for larger postoperative PTV compared to preoperative PTV in their cohort (33). Given the partly inconsistent results, the benefit of NaSRS in terms of volume reduction and consequent better protection of normal brain tissue requires further investigation. The aim of this study is to 1) compare the gross tumor volume and planning target volume of preoperative metastasis with the postoperative cavity volumes in adjuvant SRS patients, 2) identify the patient cohort with a potential volume benefit in NaSRS, and finally, 3) investigate the impact of NaSRS on NBT sparing for cases where a volume reduction is observed.

**Table 1:** Published studies on neoadjuvant SRS.

Reference	Design	Patients	Patient characteristics	Intervention	Outcome	Limitations
(22)	Combined prospective and retrospective study of preoperative SRS	47	47 patients with 51 lesions; 23 patients from database and 24 patients on prospective trial	SRS within 24 h of surgery; 80% isodose; radiation dose 14 Gy; no additional margin expansion (GTV = CTV = PTV); median follow-up time 12 months	6-, 12- and 24-month LC 97.8%, 85.6%, and 71.8%, respectively; LR more likely with lesions >10 cm ^3^ (P = 0.01) and with largest unidimensional measurement >3.4 cm (P = 0.014); DBR 38.2%; 6-, 12- and 24-month OS 7.8%, 60% and 26.9%, respectively	Retrospective character, selection bias due to lack of randomization, small patient cohort
(19)	Multicenter retrospective study to compare preoperative and postoperative SRS	180	180 patients with 189 lesions; 66 patients with 71 BM treated with preoperative SRS, 114 patients with 118 RC treated with postoperative SRS	Preoperative SRS within 48 h of surgery; postoperative SRS 2-3 weeks after surgery; 80% isodose; preoperative radiation dose 14.5 Gy and postoperative radiation dose 18 Gy; PTV of BM without additional margin expansion (GTV = CTV = PTV); margin expansion of RC defined as CTV = GTV + 1 cm and PTV = GTV + 2 cm; median follow-up time 24.6 months	No difference between groups for 1-year LR (P = 0.24), 1-year DBR (P = 0.75), and 1-year OS (P = 0.1); 2-year LMD 3.2% for preoperative SRS vs. 16.6% for postoperative SRS (P = 0.01); 2-year RN 4.9% for preoperative SRS vs. 16.4 for postoperative SRS (P = 0.01)	Retrospective study, selection bias due to lack of randomization, differences between institutions in radiographic diagnosis of RN or LMD, and criteria for recommendation of surgical intervention
(23)	Multicenter retrospective study to compare preoperative SRS and postoperative WBRT	102	102 patients with 113 lesions; 66 patients with 71 BM treated with preoperative SRS, 36 patients with 42 RC treated with postoperative WBRT	SRS within 48 h of surgery; WBRT 2-3 weeks after surgery; 80% isodose; preoperative radiation dose 14.8 Gy and postoperative WBRT with 30-37.5 Gy over 10–15 treatments; PTV of BM without additional margin expansion (GTV = PTV); median follow-up time 22.4 months	No difference between groups for 2-year-LR (P = 0.81), 2-year DBR (P = 0.66), 2-year LMD (P = 0.66) and 1-year OS (P = 0.43); crude rate of symptomatic RN 5.6% for preoperative SRS vs. 0% for postoperative WBRT (P = 0.29)	Retrospective study, selection bias due to lack of randomization, difference in duration of study arm treatments, lack of neurocognitive data
(24)	Combined prospective and retrospective study of preoperative SRS	117	117 patients with 125 lesions; 93 patients from database and 24 patients on prospective trial	SRS within 48 h of surgery; 80% isodose; radiation dose 15 Gy; PTV of BM without additional margin expansion (GTV = PTV); median follow-up time 18.7 months	2-year LR 25.1%; 2-year DBR 60.2%; 2-year LMD 4.3%; 2-year RN 4.8%; 1- and 2-year OS 60.6% and 36.7%, respectively	Retrospective study, selection bias due to lack of randomization, lack of neurocognitive, neurological death, and quality-of-life information
(25)	Retrospective study of preoperative SRS	12	12 patients	SRS within 24 h of surgery; radiation dose 16 Gy; median follow-up time 13 months	6- and 12-month LC 81.8% and 49.1%, respectively; LR 33%; DBR 67%; LMD 17%; RN 0%; 6- and 12-month OS 83.3% and 74.1%, respectively	Retrospective study, selection bias due to lack of randomization, small patient cohort, large percentage of female patients with breast cancer, therefore, results might not be generalizable
(26)	Retrospective study of preoperative SRS	19	19 patients with 22 lesions; 8 patients with previously treated recurrent lesions (previous treatment of 5 patients with SRT and 3 patients with surgical resection)	SRC within 24 h to 48 h of surgery; 80% isodose; radiation dose 18 Gy; median follow-up time 6.3 months	LR in two cases 5.5 and 17.4 months after treatment (10.5%); DBR in four cases (21.1%); LMD in one case (5.3%) 1.5 months after treatment; RN in one case (5.3%) 4.6 months after treatment; OS 89.5%	Retrospective study, selection bias due to lack of randomization, small patient cohort, short follow-up time
(27)	Retrospective study of preoperative SRS	242	242 patients with 253 lesions	SRS within 24 h of surgery; 80% isodose; radiation dose 15 Gy; PTV of 166 BM without additional margin expansion (GTV = PTV); 81 lesions with PTV = GTV + 0.5 mm or PTV = GTV + 1 mm; unknown PTV in 6 lesions; median follow-up time 24.9 months	2-year LR 17.9%; 2-year DBR 45.9%; 2-year LMD 7.6%; symptomatic adverse radiation effects 3.5%; 1- and 2-year OS 57.7% and 38.4%, respectively; subtotal resection as the primary risk factor for LR	Retrospective study, selection bias due to lack of randomization, lack of neurocognitive and quality-of-life information, potential confounding for OS endpoints
(28)	Prospective phase II dose escalation study of preoperative SRS	27	27 patients with brain metastases >2 cm in maximal dimension	Dose escalation at 3 Gy increments from currently accepted RTOG dosing; cohorts of 2–6 patients treated at each dose; SRS within 2 weeks of surgery; median follow-up time 7.4 months	No DLT; 6- and 12-month LC 93.8% and 72.3%, respectively; 6- and 12-month with DC 38.6% and 25.8%, respectively; LMD in one patient 5 months after SRS; symptomatic adverse radiation effects 15%; 6- and 12-month OS 80.8% and 53.5%, respectively	NR
(29)	Retrospective study of preoperative SRS	28	28 patients with 29 lesions after exclusion of nonmetastatic pathology; hypofractionated SRS used in 18 lesions and single-fraction SRS in 11 lesions; 12 patients from database and 17 patients on prospective trial	SRS within 24 h of surgery; hypofractionated SRS with 24 Gy in 3 fractions and single-fraction SRS with 20 Gy; PTV of BM defined as PTV = GTV + 1 mm; median follow-up time 12.8 months	1-year LC 91.3%; 1-year DC 51.5%; 1-year LMD 4%; 1-year RN 5%; 1-year OS 60.1%;	Case series, selection bias due to lack of randomization, small and heterogeneous patient cohort

BM, brain metastasis; CTV, clinical target volume; DBR, distant brain recurrence; DC, distant control; DLT, dose-limiting toxicity; GTV, gross tumor volume; LC, local control; LMD, leptomeningeal disease; LR, local recurrence; NR, not reported; OS, overall survival; PTV, planning target volume; RC, resection cavity; RN, radio necrosis; SRS, stereotactic radiosurgery; SRT, stereotactic radiotherapy; WBRT, whole brain radiotherapy.

## 2 Methods

### 2.1 Patient cohort

**Figure 1:**
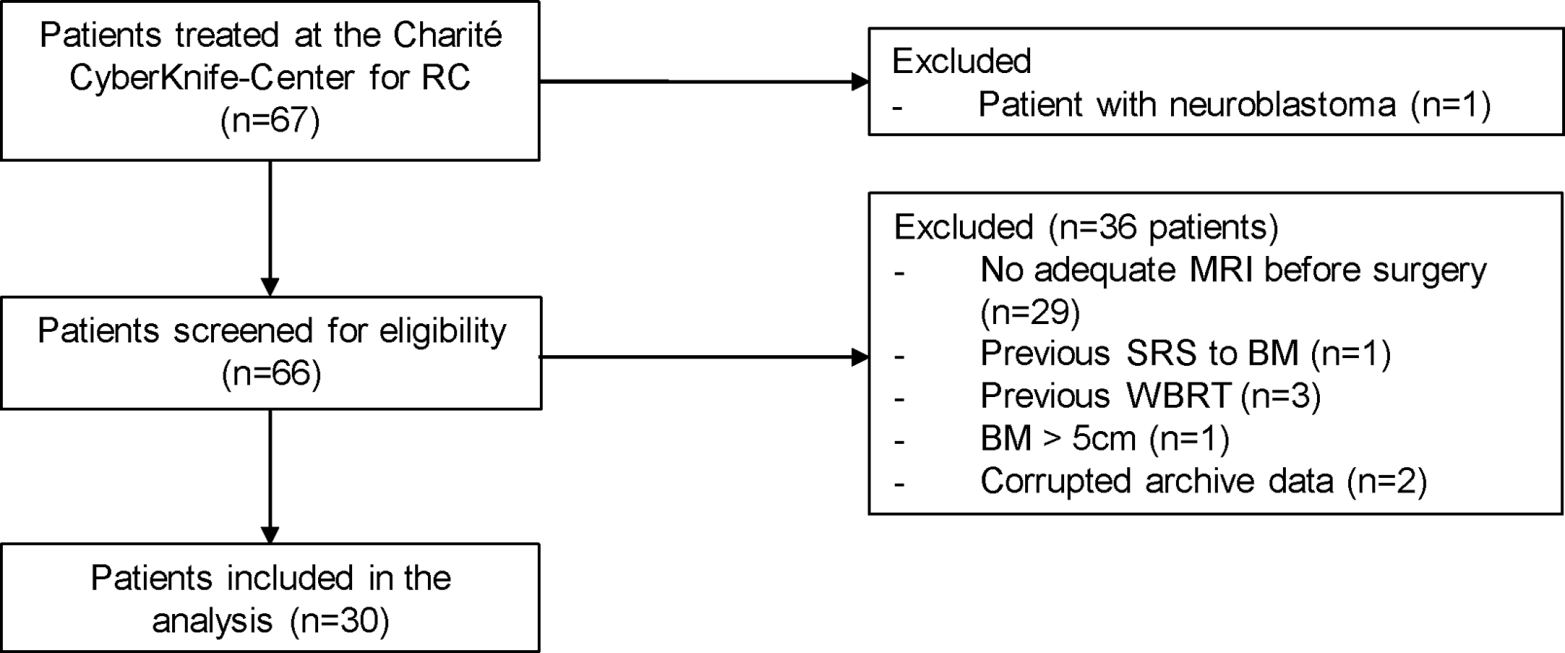
Patients with surgically resected brain metastases who underwent postoperative stereotactic radiosurgery to the resection cavity in our department were included. One pediatric patient with neuroblastoma was excluded from the cohort. Additional exclusion criteria included: no adequate intracranial MRI before surgery, previous SRS at the index lesion or WBRT as well as a diameter of the preoperative lesion greater than 5 cm. Two patients had to be additionally excluded because their data could not be extracted from the Accuray archive. RC, resection cavity; BM, brain metastasis; SRS, stereotactic radiosurgery.

This retrospective analysis of patient data and the registry of prospective patient data collection were approved by the local ethics committee, as this cohort contains both data sets (EA1/037/20). Patients in the prospective cohort signed a consent. We identified all patients with post-SRS treatment of resection cavities from brain metastases (index lesion) between July 2011 and August 2021 at our institution. We then checked whether adequate preoperative magnetic resonance imaging (MRI) was available and set a maximum diameter of 5.0 cm as the limit for the index lesion to be suitable for NaSRS simulation (34). We excluded patients with previous SRS to the index lesion or WBRT (**Figure 1**).

We collected data on patient characteristics regarding the primary disease, tumor location, morphology, operation technique (en bloc vs. piecemeal), and local and distant tumor control. Tumors were described as superficial or deep based on their location and classified as cystic or non-cystic depending on their morphology, as described previously (11, 35). The extent of resection was assessed when a postoperative MRI performed within 30 days after surgery was available, since an early MRI was not a routine neurosurgical procedure in the past years. Data on systemic treatments were also collected, but we limited the report to “yes” or “no” and, if yes, only the timing of treatment, as this was outside the scope of this project.

### 2.2 Cyberknife SRS of the resection cavities: Retrospective treatment description

The indication for post-SRS/HF-SRS treatment was decided by a multidisciplinary neuro-oncology board team including a radiation oncologist and a neurosurgeon. The Cyberknife radiosurgery treatment preparation and planning were similar to the already published algorithm of our clinic (36). Briefly, a thermoplastic mask was individually produced for each patient for treatment immobilization before contrast enhanced high-resolution thin-slice (0.75 mm) computed tomography (CT). This reference CT was co-registered to T1-weighted magnetic resonance images (MRI). Since the visible tumor in the preoperative contrast enhanced T1-weighted MRI imaging is referred to as gross tumor volume (GTV), we referred to the contour of the resection cavity as post-GTV despite the absence of tumor volume to allow for better comparability in further analyses. In case of cystic lesions, the tumor-associated cyst was included in the GTV. The post-GTV was defined as the resection cavity volume based on postoperative contrast enhanced planning CT and a co-registered T1-weighted MRI, considering the surgical pathway and meninges near the craniotomy. The post-GTV was extended by 0 to 3.0 mm at the discretion of the treating radiosurgeon to create the postoperative planning target volume (post-PTV) in this retrospective cohort (**Table 2**). In 9 out of 30 postoperative cavities no margin was added to post-GTV, therefore, in these cases post-GTV and post-PTV do not differ. The doses were mostly prescribed to the 80% isodose line covering the PTV (**Table 2**). Depending on the vicinity to the organs at risk (e.g., optic nerves, chiasm, and brainstem) and the size of the resection cavity, different dose schedules were applied. If a brain metastasis was eloquently located (e.g. in the brainstem or along the optic pathway), either a reduction of the single fraction dose or hypofractionation was performed, depending on how the dose constraints were met. Briefly, doses in the range of 15–19 Gy, 21–24 Gy, and 25–30 Gy have been applied for one, three, and five fractions, respectively (**Tables 2**, **3**).

**Table 2:** Treatment characteristics of the performed resection cavity irradiation as well as gross tumor- and planning target volumes in the hypothetical planning.

Pat. Nr.	Pre-GTV (cm^3^)	Pre-PTV (cm^3^)	Post-GTV (cm^3^)	Post-PTV (cm^3^)	Post-Margin* (mm)	Std. Post-PTV (cm^3^)	Min Dose (Gy)	Max Dose (Gy)	Mean Dose (Gy)	Prescribed Dose (Gy)	Fx	nCI **	Coverage (%)	Isodose
1	2.6	3.6	6.0	6.0	0	10.2	17.0	22.5	20.5	18.0	1.0	1.1	98.9	80.0
2	2.9	3.9	6.5	9.7	1.5-2.5^+^	10.8	16.1	22.5	20.5	18.0	1.0	1.0	97.5	80.0
3	3.0	3.9	3.2	5.5	1.5	6.3	17.7	24.9	22.2	19.0	1.0	1.1	99.5	80.0
4	3.1	4.1	1.3	1.3	0	3.1	22.8	30.0	26.4	24.0	3.0	1.3	98.9	80.0
5	3.8	5.0	3.3	5.7	2.0	6.0	19.6	26.3	23.5	21.0	3.0	1.2	98.2	70.0
6	4.5	5.9	9.7	14.4	2.0	14.4	14.2	18.7	16.9	15.0	1.0		97.7	80.0
7	5.5	7.0	8.3	15.3	2.8	12.9	20.9	30.0	27.3	24.0	3.0	1.1	97.7	80.0
8	5.9	7.9	11.1	16.6	1.5-3.5^+^	17.2	23.2	30.0	27.4	24.0	3.0	1.1	99.7	80.0
9	6.7	8.0	9.6	13.0	1.5	15.8	26.4	37.5	34.1	30.0	5.0	1.1	99.1	80.0
10	7.2	11.7	6.5	6.5	0	10.7	17.1	22.6	20.7	18.0	1.0	1.2	99.3	80.0
11	8.2	10.4	26.7	31.6	1	38.3	26.3	37.5	34.0	30.0	5.0	1.0	95.1	80.0
12	8.2	10.7	7.1	7.1	0	11.3	22.6	30.0	27.6	24.0	3.0	1.1	98.6	80.0
13	8.6	9.9	5.2	7.4	1.5	9.1	20.0	26.1	23.8	21.0	3.0		98.1	80.0
14	8.9	10.2	4.0	5.8	1.5	7.4	22.9	30.1	27.6	24.0	3.0	1.1	99.7	80.0
15	9.9	12.1	8.7	14.4	2	14.1	21.2	30.0	27.0	24.0	3.0	1.1	97.2	80.0
16	10.0	12.6	7.7	12.2	2	12.4	19.9	24.7	23.2	21.0	3.0	1.1	99.9	80.0
17	10.4	12.6	21.8	28.7	1.5-2.5^+^	30.6	21.6	26.9	24.9	22.5	3.0		99.3	85.0
18	11.6	13.3	13.4	17.4	1.5	20.7	22.8	30.0	27.5	24.0	3.0	1.1	99.7	80.0
19	11.6	17.3	8.9	15.3	2.1	14.7	6.8	18.0	14.4	14.4^#^	1.0	1.1	60.8^#^	80.0
20	12.2	15.4	14.5	14.5	0	21.4	22.1	30.0	27.3	24.0	3.0	1.1	97.5	80.0
21	13.3	21.8	15.1	15.1	0	24.3	14.4	20.0	17.8	16.0	1.0	1.1	95.2	80.0
22	13.4	16.5	13.5	13.5	0	19.8	21.2	30.0	27.0	24.0	3.0	1.1	97.4	80.0
23	16.0	19.0	21.5	21.5	0	30.1	11.9	20.7	17.7	15.0	1.0	2.0	95.1	70.0
24	20.6	25.0	30.9	36.0	1	42.9	24.1	37.5	33.9	30.0	5.0	1.0	93.8	70.0
25	20.6	24.2	13.4	18.6	1.5	20.5	30.0	40.6	37.1	32.5	5.0	1.0	97.7	80.0
26	21.0	30.2	16.8	22.8	1.8	25.0	21.8	30.3	27.7	24.0	3.0	1.1	98.5	80.0
27	21.4	25.6	33.9	45.7	2	45.9	23.2	35.7	30.3	25.0	5.0	1.1	99.73	70.0
28	24.4	28.5	9.2	9.2	0	14.8	21.7	30.0	27.4	24.0	3.0	1.1	98.1	80.0
29	32.8	35.9	17.1	22.2	1.5	26.0	19.5	26.3	23.6	21.0	3.0	1.1	98.5	80.0
30	34.5	40.1	9.1	14.4	2	14.6	22.9	30.0	27.6	24.0	3.0	1.1	99.6	80.0

*As the exact margins were not documented separately, a retrospective approximation was performed based on the GTV and PTV volumes. ^+^ The actual margin was too irregular to be determined retrospectively., **In cases where other brain metastases were irradiated at the same session as the resection cavity the conformity index for the resection cavity was not documented separately., #This plan was interrupted so that the prescribed dose could not be administered and ended at 14.4 Gy, so this coverage was not included into the results., Fx, fractions; GTV, gross tumor volume; pre, preoperative; nCI, new conformality index; PTV, planning target volume std, standardized.

The margin added to the pre-GTV was set to 1 mm, while this was 2 mm for the standardized post-PTV.

**Table 3:** Patient, tumor and treatment characteristics.

Number of patients Number of resection cavity treated lesions	30 30
Sex, n (%) Female Male	14 (46.6%) 16 (53.3%)
Age Average (SD) Median (Range)	63.1 (13.3) 66.9 (32.1-85.7)
Primary tumor, n (%) NSCLC Breast cancer Malignant melanoma RCC Rest	17 (56.7%) 4 (13.3%) 2 (6.7%) 2 (6.7%) 5 (16.7%)
Localization of brain metastases, n (%) Occipital Frontal Cerebellar Temporal Parietal Intraventricular	7 (23.3%) 7 (23.3%) 5 (16.7%) 6 (20%) 4 (13.3%) 1 (3.3%)
Dose and fractionation, n (%) 14.4 Gy in 1 fraction* 15 Gy in 1 fraction 16 Gy in 1 fraction 18 Gy in 1 fraction 19 Gy in 1 fraction 21 Gy in 3 fractions 22.5 Gy in 3 fractions 24 Gy in 3 fractions 25 Gy in 5 fractions 30 Gy in 5 fractions 32.5 Gy in 5 fractions	1 (3.3%) 2 (6.7%) 1 (3.3%) 3 (10%) 1 (3.3%) 4 (13.3%) 1 (3.3%) 12 (40%) 1 (3.3%) 3 (10%) 1 (3.3%)
Fractionation regime, n (%) HF-SRS S-SRS	22 (73.3%) 8 (26.6%)
Extent of resection, n (%) Gross total Subtotal n.a.	22 (84.6%**) 4 (15.4%**) 4 (13.3%)
Method of resection, n (%) En bloc Piecemeal n.a.	6 (20%) 14 (46.7%) 10 (33.3%)
Time interval between resection date and irradiation Average (SD) Median (Range)	36.7 days (10.4) 37.5 days (17-57)

HF-SRS, hypofractionated SRS; NSCLC, non-small cell lung cancer; RCC; renal cell carcinoma; Rest = gastrointestinal, ovarian cancer, cervical cancer, base of tongue, cancer of unknown origin; s-SRS, single-fraction SRS. *This plan was interrupted so the prescribed dose could not be applied and ended at 14.4 Gy. **calculated as % of 26 patients with the available information.

The isodose volume of the normal brain tissue (NTB, excluding the PTV), circumscribed with 10.0 Gy (V10 < 10 cm^3^) for single fraction, 18.0 Gy (V18 < 10 cm^3^) for three fractions, and 28.8 Gy (V28.8 < 7 cm^3^) for five fractions, which were defined based on the published data on this topic, was measured and recorded in each patient as clinical routine to determine the risk of adverse effects on the surrounding healthy brain (37–39). However, if the parameters defined in the internal guidelines were not applicable, i.e., due to a dose reduction to 25 Gy in 5 fractions, the evaluated parameter was adjusted individually (**Table 2**: Pt. number 27, **Table 4**: case 7, the evaluated parameter adjusted to V22.5 Gy < 10 cm³ accordingly). To protect the other organs at risk, we applied the recommended threshold doses published by Benedict et al. for SRS/HF-SRS in particular (40).

**Table 4:** Simulation planning details.

CASE	Fx	Dose	Pre-GTV (cm^3^)	Post-GTV (cm^3^)	Change in GTV (post-GTV - pre-GTV) (cm^3^)	Brain PTV Volume Parameter (Vxx)	Volume of the defined DVH parameter (pre) (cm³)	Volume of the defined DVH parameter (post) (cm³)	Percentual difference of the dose defined DVH parameter (pre/post)
1	1	18	4.5	9.7	5.2	10.0	24.6	36.4	68%
2	3	24	5.5	12.9	7.4	18.0	5.8	11.0	52%
3	3	24	5.9	11.1	5.1	18.0	9.0	12.5	72%
4	5	30	8.2	26.7	18.6	28.8	1.0	2.0	47%
5	3	22.5	10.4	21.8	11.4	18.0	15.1	22.1	68%
6	5	30	20.6	30.9	10.3	28.8	2.4	7.1	33%
7*	5	25	21.4	33.9	12.6	22.5	12.0	14.3	84%

DVH, dose volume histogram; Fx, fractions; GTV, gross target volume; pre, preoperative; PTV, planning target volume. *Due to the dose reduction to 25 Gy in 5 fractions, the DVH parameter was defined as V22.5.

The equivalent dose for 2 Gy per fraction was calculated according to the LQ-model assuming an α/β ratio 10 for tumor (EQD2_10_) for the comparison to conventional irradiation treatment. The calculated EQD2_10_ encompassing the PTV was 31.2–45.9 Gy for a single fraction, 29.8–36.0 Gy for three-fraction treatment, and 31.2–40.0 Gy for five-fraction SRS.

### 2.3 Follow-up

Radiological imaging by contrast-enhanced MRI and clinical assessment were performed every 3 months as follow-up. The latest available follow-up was included in this analysis. MRI scans were interpreted by both a radiology specialist and the radiosurgery physician to determine response to treatment. We first examined local and distant brain control. Local recurrence was defined as a new progressive nodular contrast enhancing lesion involving the resection cavity as performed in other studies (27, 29) or when a progressive residual lesion with a diameter increase by at least 20.0% was observed (41). Distant failure was defined as new brain metastasis elsewhere or as LMD on the follow-up MRI. LMD is also reported separately to differentiate these cases from the patients with only new solid lesions. The complications were recorded based on Common Terminology Criteria of Adverse Events CTCAE Version 5.0. 

### 2.4 Simulation contouring and planning study

After co-registration of the preoperative MRI with the planning reference CT, the unresected metastasis was countered first as pre-GTV based on the contrast enhanced T1 weighted thin-sliced MRI. Clinical target volume was equal to pre-GTV. When creating the pre-PTV, a standardized margin of 1 mm was added to all metastases in accordance with hospital guidelines, which is commonly used to compensate for uncertainties (42). The post-PTV was taken from the original plans. The hypothetical preoperative volumes (pre-GTV and pre-PTV, respectively) were subsequently compared with the real postoperative irradiation volumes (post-GTV and post-PTV, respectively; **Table 2**). In addition, a standardized post-PTV (std. post-PTV) volume was generated with a 2 mm margin, as the latest practice guidelines from international stereotactic radiosurgery society recommend a margin of 2 to 3 mm (32), while the retrospective cohort was heterogenous in this regard. The volume changes were assessed in absolute values (cm^3^) and also in percentage of volume difference as described by Atalar et al. (31).

For the cases with a GTV volume increase greater or equal to 5 cm^3^ from the pre-GTV to the post-GTV, a retrospective simulation study was performed, identifying the potential dose sparing for normal brain tissue. One patient had to be excluded due additional multiple lesions in the treated clinical treatment plan, which strongly influence the exposure of NTB. Within this simulation, first all clinical existing patient plans on the post-PTV were newly optimized within the current available treatment planning system (Precision 3.1 software, Accuray Inc., Sunnyvale, CA, USA). In the second step, the identical plan templates were used for a new optimization on the pre-PTV with 1 mm margin to GTV, therefore, simulating the equivalent dose prescription to the pre-PTV as employed for the clinical used post-PTV treatment plan. Subsequently, to guarantee equivalent sparing of the organs at risk, the weights of conformity ensuring margins around the targets were tightened until differences in PTV coverage were within 0.2%. For the evaluation of dose distribution for both existing plans a healthy brain tissue (brain minus pre-PTV/brain minus post-PTV) was generated and the clinically employed dose-volume histogram (DVH) parameter evaluated. The relative effect of the NaSRS was evaluated in terms of the dose-specific DVH parameter ratio between simulated NaSRS and postoperative original irradiation (pre/post), whereas a value below 100% depicts a relative decrease and above 100% a relative increase of the evaluated DVH parameter.

### 2.5 Statistics

The data are presented as mean, standard deviation, median, and range depending on the context. As the volumes before and after GTV and PTV were not normally distributed in the Kolmogorov-Smirnov test, we performed the Wilcoxon test to compare paired data and the Mann-Whitney-U-test for unpaired data. A correlation was established between pre-GTV and changes in GTV as well as PTV volumes and a correlation analysis was also performed between the time from surgical resection to post-GTV MRI acquisition in days and GTV volume change (Pearson correlation). Progression-free survival was investigated using Kaplan-Meier analysis for local and distant control as well as LMD-free survival. Furthermore, overall survival was also calculated using Kaplan-Meier analysis based on information from the Berlin-Brandenburg tumor registry. Patients are censored when follow-up is terminated prior to an event. To identify possible predictors of post-GTV volume change, we performed a multiple linear regression analysis. We defined the dependent factor as GTV change (normally distributed) and assessed the morphologic characteristics of the tumor such as cystic/non-cystic, superficial/deep, and supratentorial/infratentorial (categorical), as well as pre-GTV volume as variables. IBM SPSS Statistic Program (Version 25.0. Armonk, NY: IBM Corp.) was used, and P ≤ 0.05 was considered as statistically significant. Prism 9 was used for the graphical representation of the collected data. Since this was only an exploratory study, no correction for multiple testing was performed.

### 2.6 Literature review

**Figure 2:**
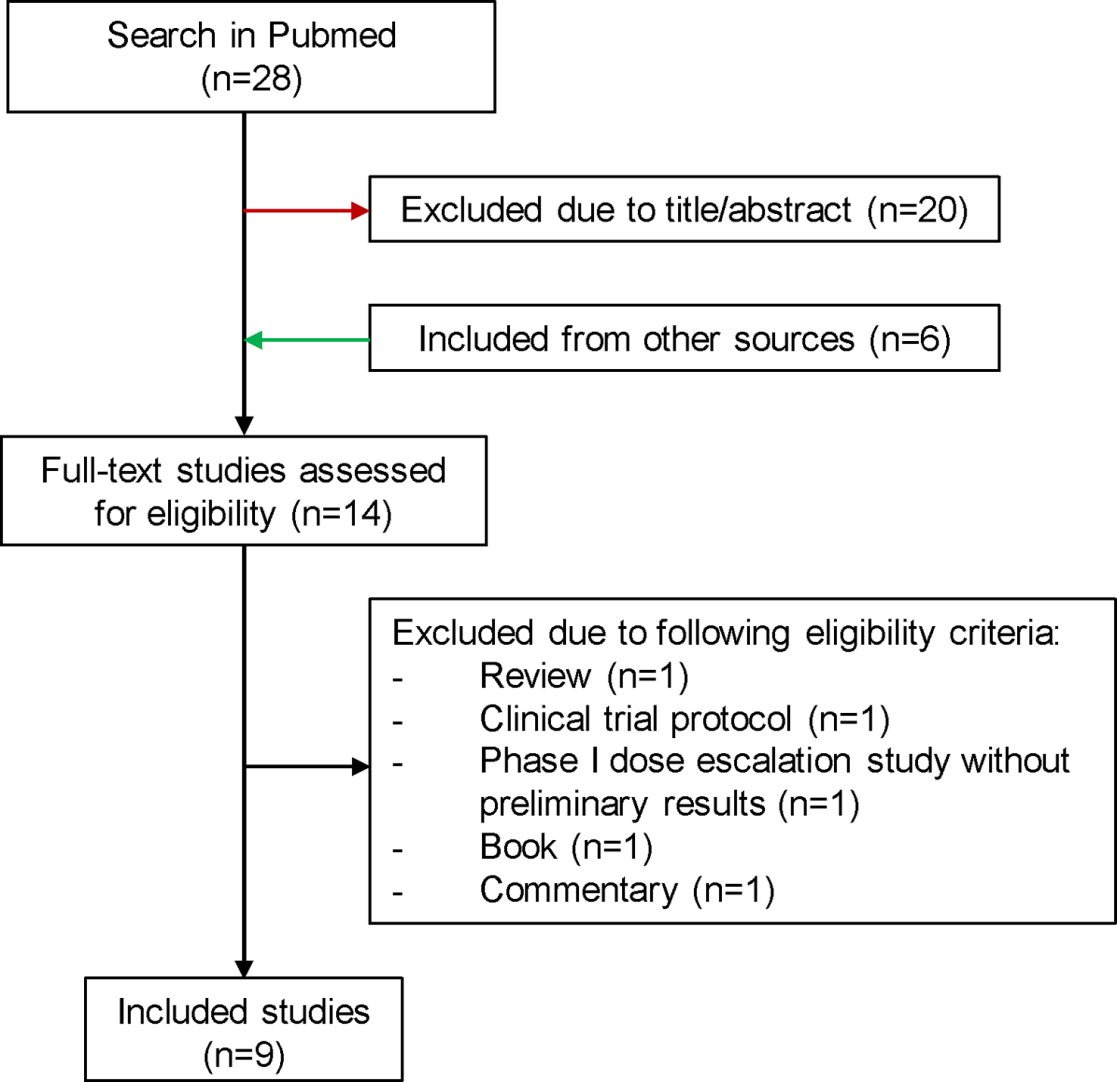
Flowchart for the selection process of published studies for preoperative SRS. SRS, stereotactic radiosurgery.

Since the latest reviews were not up to date, we have created an overview of published and ongoing NaSRS studies (24, 43). For the literature review the electronic database “PubMed” was consulted on the 27^th^ of July 2022 according to PRISMA guidelines. The search included the following terms: “((neoadjuvant [MeSH Terms]) AND (radiosurgery [MeSH Terms]) AND (brain metastases [MeSH Terms])”. After excluding studies due to unrelated title/abstract and including publications from other sources, a total of 14 full-text studies were assessed for eligibility (**Figure 2**). We summarized 9 studies in **Table 1**. For the ongoing studies on NaSRS, the U.S. National Library of Medicine (clinicaltrials.gov) and the WHO Clinical Trials Registry Platform (trialsearch.who.int) databases were searched on 18th August 2022. For the U.S. National Library of Medicine, the advanced search mode was used. The following entries were applied: Condition: Brain Metastases; Intervention: (Neoadjuvant OR Preoperative) AND Radiosurgery. For the WHO Registry, the following term was used: Radiosurgery AND Brain Metastasis AND (Neoadjuvant OR preoperative). A total of 21 results were found in both databases. After accessing the results, one study was excluded because it did not include SRS as a neoadjuvant treatment (**Supplementary Table 1**).

## 3 Results

### 3.1 Patient cohort

We identified 30 resection cavity patients fulfilling the criteria in our retrospective cohort (**Figure 1**). Demographic and clinical characteristics are summarized in **Table 3**. Briefly, gender was evenly distributed (males 53.3%, females 46.6%), mean age was 63.1 years with a range of 32.1 to 85.7 years. The most frequent primary tumor type was lung cancer (56.7%) followed by breast cancer (13.3%), malignant melanoma, and renal cell carcinoma (RCC) with 6.7% each. Only 16.7% were infratentorial. The superficial localization was more frequent with 73.0%, while 60.0% were cystic tumors. Gross total resection was achieved in 84.6% of cases, and the piecemeal technique was most frequently used (**Table 3**). In 9 patients a postoperative MRI within 48 hours was available. In the cases with presumably subtotal resection the median time interval between the first MRI after resection was 26 days (range: 20-27 days).

### 3.2 Resection cavity SRS details and treatment response

The median time interval between surgery and SRS/HF-SRS was 37.5 days with a range of 17-57 days. In 26.6% of the cases, the treatment was performed in a single fraction as SRS with a median prescribed dose of 18.0 Gy (**Tables 2**, **3**). In more than half (56.9%), a multi-session treatment as HF-SRS with 3 fractions was preferred, while 5 fractions were less frequent with 16.6%. For one patient the clinical treated dose was reduced to 25 Gy in 5 fractions due to additional sequentially irradiated lesions. Coverage ranged between 93.8% to 99.8%. Further treatment details are listed in **Table 2**. Information on systemic treatments was present in 29 patients. Additional systemic treatment with chemotherapy and/or immunotherapy and/or targeted therapies was performed in a total of 26 patients. In each 27.6% of these patients, this treatment took place before or during irradiation, in 24.1% before and after treatment. Only in three cases did the systemic treatment take place exclusively after irradiation.

**Figure 3:**
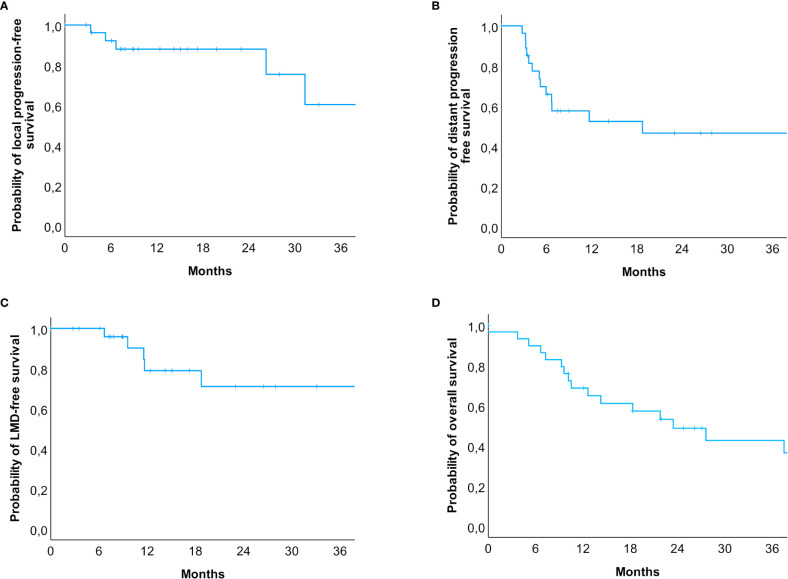
Kaplan-Meier-Curves for **(A)** local progression-free, **(B)** distant progression-free, **(C)** leptomeningeal disease-free survival and **(D)** overall survival. **(A)** Patients at risk were n = 30 (0 months), n = 23 (6 months), n = 15 (12 months), n = 7 (24 months), and n = 3 (36 months). **(B)** Patients at risk were n = 30 (0 months), n = 17 (6 months), n = 10 (12 months), n = 6 (24 months), and n = 4 (36 months). **(C)** Patients at risk were n = 30 (0 months), n = 25 (6 months), n = 14 (12 months), n = 7 (24 months), and n = 4 (36 months). **(D)** The estimated median survival of our patients was 23.4 months (95% CI: 11.3 – 35.6). Patients at risk were n = 30 (0 months), n = 19 (12 months), n = 11 (24 months), n = 7 (36 months). In total, 14 events occurred within 24 months after the radiosurgical treatment, but only 4 events in the subsequent years were reported.

A total of 27 patients were re-examined with a median follow-up time of 14.2 months (range: 2.8 to 72.7 months). Within this period, local progression occurred in five patients (18.5%), whereas distant intracranial progression occurred in 51.8% (n = 14). LMD occurred in 6 cases (22.2%), and all six patients also had lesional distant recurrences. In three cases, SRS treatment was administered, and the other three cases occurred after HF-SRS (two received three fractions and one received five fractions). The estimated rates for local progression-free survival at 6.0, 12.0, and 24.0 months were 92.7%, 88.0%, and 88.0%, respectively (**Figure 3D**), while the rates for distant progression-free survival at 6.0, 12.0, and 24.0 months were 67.9%, 54.3%, and 47.5%, respectively. The estimated LMD-free survival rates were accordingly 100.0%, 81.4%, and 72.4% (**Figures 3A–C**). The estimated median overall survival was 23.4 months (95% CI: 11.3 – 35.6). The overall survival rates at 12.0, 24.0 and 36.0 months were 69.0%, 49.3%, and 44.1%, respectively (**Figure 3D**). Imaging-based suspected radionecrosis was observed in two cases (7.4%). A total of five adverse effects were recorded, a local alopecia and mild headache as CTCAE grade I and moderate dizziness, aphasia, and severe headache as grade II.

### 3.3 Comparison of volumes

**Figure 4:**
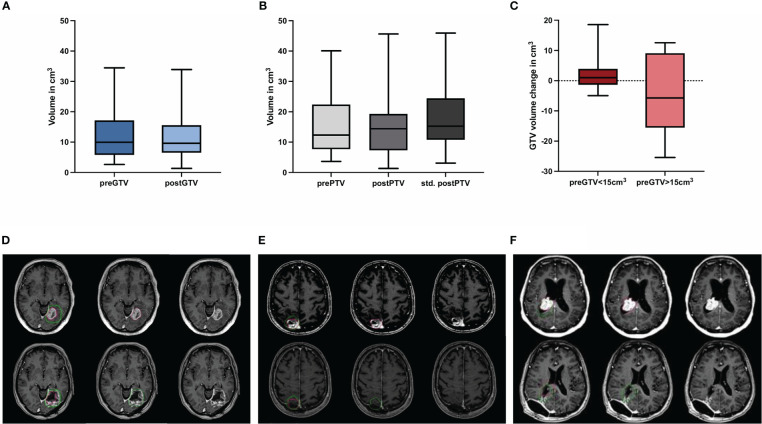
Quantification of the preoperative (pre) and postoperative (post) **(A)** gross tumor volume (GTV) and **(B)** planning target volume (PTV) including hypothetical standardized PTV with a 2 mm margin to post-GTV (n = 30; no significant differences, Wilcoxon test), **(C)** shows the volume change of binarized pre-GTV volumes depending on size with a cut-off of 15 cm^3^ (n = 22 for pre-GTV < 15.0 cm^3^ and n = 8 for pre-GTV > 15.0 cm^3^; no significant differences, Mann-Whitney-U-test.) Boxplots represent the interquartile range, the thicker line inside the boxes the median, and the whiskers indicate the range from minimum to maximum. Representative case presentations with one deep **(D)** one superficial **(E)** and one intraventricular **(F)** metastasis from non-small cell lung carcinoma shown in axial MRI images with contrast demonstrating comparison of GTV in red for preoperative metastases and in green for the resection cavity. In these cases, an increase in post-GTV compared with pre-GTV can be seen, which was 227.3% in **(D)**, 86.6% in **(E)**, and 19.3% in **(F)**.

**Figure 5:**
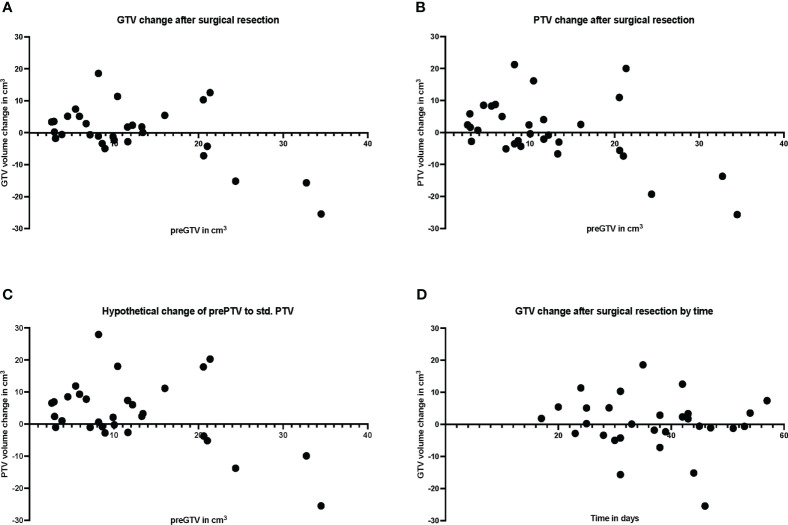
Plot of individual patient data (n = 30) for resection cavity volume changes compared with preoperative volumes, **(A)** for gross tumor volume (GTV), **(B)** for planning target volume (PTV), and **(C)** for standardized PTV, shown relative to preoperative (pre) GTV. **(D)** shows the volume change of the resection cavity after surgical resection in relation to the days between surgery and MRI.

The pre-GTV and pre-PTV are shown in **Figures 4A–F** and in **Table 2** in comparison to the postoperative values. Here, we could not find any significant differences between pre- and postoperative volumes when looking at the median values (**Figure 4A**, P = 0.551 and B, P = 0.781), while the median of std. post-PTV tended to be higher than pre-PTV (P = 0.051). The volume change in binarized pre-GTV volumes depending on size with a cut-off at 15.0 cm^3^ highlighted the wider range in larger tumors, while the smaller tumors tended to have greater GTV after resection, but this did not reach significance (**Figure 4C**, P = 0.205). We also present the distribution of postoperative volume changes as an absolute value in cm³ compared to the original tumor size (pre-GTV) (**Figures 5A, B**). Remarkably, the majority of cases with enlargement greater than 5.0 cm^3^ were smaller tumors with pre-GTV < 15.0 cm^3^ (62.5% of all cases with > 5 cm^3^), whereas larger tumors greater than 25 cm^3^ showed only a decrease in post-GTV and post-PTV. For the hypothetical standardized PTV with the fixed margin of 2 mm, the difference from pre-PTV was greater for the few cases with no or less margin in the original post-PTV (**Figure 5C**). The correlation assessment revealed a significant negative correlation between pre-GTV volume and all three volume changes (GTV change: r: -0.558, P = 0.001, PTV change: r = -0.507, P = 0.004, hypothetical PTV change: r = -0.451, P = 0.012). As it was shown that the time after surgery may influence the size of the resection cavity (44), we analysed the volume change in GTV in relation to the time interval between surgery and MRI in our cohort. However, we could not identify a significant correlation (r: -0.091, P = 0.634, **Figure 5D**).

### 3.4 Predictors for GTV change

A multiple linear regression was calculated to predict a volume change of GTV after resection. A significant regression equation was found (F (4, 25) = 3.060, P = 0.035) with an adjusted R^2^ = 0.221 (unadjusted R^2^ = 0.329). The pre-GTV size was the only significant predictor for volume change of GTV (**Table 5**).

**Table 5:** Multiple linear regression analysis for prediction of GTV change.

Variables	B	95% CI	Beta	t	P
superficial vs. deep	2.2	-4.4 - 8.8	0.1	0.7	0.490
cystic vs. not cystic	-1.2	-7.3 – 4.9	-0.1	-0.4	0.694
supra- vs. infratentorial	-0.6	-8.8 - 7.3	0.02	-0.2	0.878
pre-GTV in cm	-0.6	-0.9 - -0.2	-0.6	-3.2	0.004

Dependent Variable: GTV change (pre-GTV to post-GTV). R^2^ adjusted: 0.221 (n = 30, P = 0.035). CI, confidence interval for B; GTV, gross tumor volume; post-GTV, postoperative GTV; pre-GTV, preoperative GTV.

### 3.5 Simulation planning study

**Figure 6:**
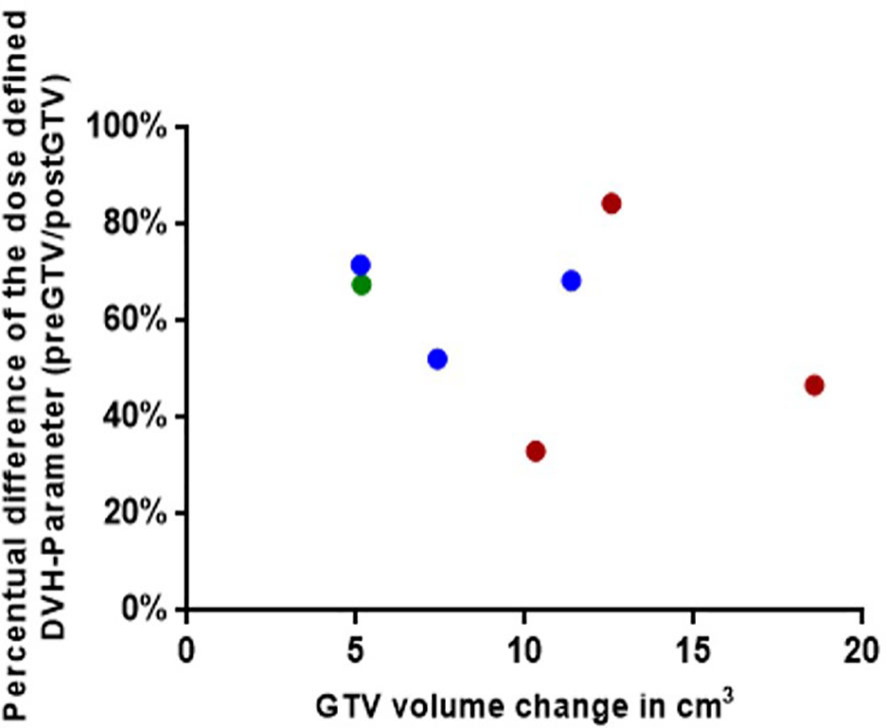
Graphical representation for the seven simulated plannings, visualizing the dose-defined dose-volume histogram (DVH) parameter ratio between simulated NaSRS and postoperative original irradiation (pre/post) against the absolute difference in GTV volume. Fractionations are color coded: Single fraction in green, 3-fractions in blue, and 5-fractions in red. GTV, gross tumor volume.

We identified a total of 16 patients with post-GTV greater than pre-GTV, however, the range for the GTV change was wide, from 0.11 to 18.57 cm^3^; thus, we set the cut-off at 5 cm^3^ based on the median GTV increase of 4.3 cm^3^. Within the planning study for both treatment scenarios clinically applicable treatment plans could be generated for 7 identified patient cases with an increase of GTV from the pre-GTV to the post-GTV of over 5 cm³. The median annotated GTV of the primary metastasis was 8.2 cm³ with a range of 4.5–21.4 cm³. The median increase of GTV was 5.5 cm³ with a range of 5.1–18.6 cm³. In this cohort, margins for post-PTV were heterogeneous 0 to 3 mm, as in the entire cohort, however, all but one had margins of 1 to 3 mm. In the hypothetical planning NBT exposure was less in NaSRS group with a median of only 67.6% (range: 33.2–84.5%) of NBT calculated with post-PTV receiving the fractionation-specific evaluated dose. The relative NBT exposure in relation to the change in GTV volume is shown in **Figure 6**. Accordingly, the evaluated DVH parameter showed a median relative decrease for the analyzed brain minus PTV parameter of 32.4% with a range of 15.5–66.9% (**Table 4**). The analyzed Pearson correlation coefficient for the changes in volume in relation to the relative decrease of the evaluated DVH parameter presented no significant correlation (r = -0.16; P = 0.73).

## 4 Discussion

In this study, we show an increase in the volume of the resection cavity dependent on the initial tumor size, which may lead to higher dose exposure of the NBT in selected cases. In addition, we provide an update on published and ongoing NaSRS studies as a comprehensive overview and discuss crucial aspects for the further use of NaSRS.

Neoadjuvant SRS or so-called preoperative SRS has become a hot topic in the treatment of brain metastases requiring surgery with potential benefits such as better local control, less LMD, and more convenient target delineation. NaSRS was included in the most recent American Society of Radiation Oncology (ASTRO) 2022 guidelines with a conditional recommendation as a potential alternative to post-SRS, but the level of evidence was rated low by the endorsement panel, warranting further study on this topic (45). **Table 1** lists 9 studies that have been published on NaSRS, including the most recent studies following the latest reviews (24, 43). Although the existing data are mainly from retrospective studies, they have led to the initiation of several prospective studies that are currently underway (**Supplementary Table 1**). Our department also takes part in a Phase II bicentric study with the University of Toronto (NCT03368625) (34). Per protocol, the diameter of the index lesion is set between 2.0 to 5.0 cm, and NaSRS administered at a single dose of 14.0 to 20.0 Gy depending on tumor volume. In example, 14 Gy was the dose regimen for tumors with a volume of ≥ 20 to < 50 cc.

However, the protocols of ongoing studies differ in terms of dosing; in particular, a dose-escalation study aims to determine the maximum tolerated dose of SRS administered before neurosurgical treatment, whereas a dose de-escalation study compares 12.0 to 15.0 Gy. In addition, there are several studies testing HF-SRS (**Supplementary Table 1**). Because the results of several prospective studies are still outstanding to clarify the role of NaSRS in routine clinical practice, we sought to determine its potential benefits in a matched analysis based on hypothetical planning of preoperative lesions treated after surgery in real-world settings. There are several studies that have investigated resection cavity dynamics independent of association with NaSRS, which are summarized in the review by Yuan et al. (44). Here, the authors reported that on average the resection cavities were smaller than the preoperative tumors. The most postulated predictor of greater volume depletion after surgery was larger tumor size (31, 46, 47), whereas Scharl et al. made an inverse observation (48). In our study, we found a negative correlation between pre-GTV size and resection cavity volume change, with smaller tumors leading to more changes, often an increase in post-GTV like Atalar et al. (31). Our data suggested 15.0 cm^3^ as a possible cut-off volume to predict a volume increase, however, this must be assessed in larger cohorts. In comparison, Atalar et al. reported that for pre-resection tumors greater than 4.2 cm^3^ the cavity volume was smaller than the tumor itself (31). In this context, one may ask why surgery is necessary at all for small lesions. Surgery for smaller lesions that can be treated directly with SRS is still warranted in selected cases due to severe edema, neurologic symptoms, and histologic tissue demands. Steindl et al. published recently a large series including 1608 patients with NSCLC brain metastases. Although they did not include tumor volumes in the investigation, it is of importance to note that 740 of 1107 (68.8%) patients with tumors less than 3 cm in diameter suffered from neurologic symptoms (49). In addition, the potential discordance between primary tumor and CNS metastases, as demonstrated in several publications, necessitates in selected cases a surgical tissue sampling to optimize systemic treatment (50–52). Another inconsistent aspect is the resection cavity dynamics over time as shown in the above-mentioned review with different observations amongst seven studies (44). In our series, we could not find any correlation between the time interval from surgery to postoperative planning MRI and volume change consistent with Atalar et al. (31). Importantly, to exclude residual tumor after resection an early MRI must be performed within 48 hours (53). However, given that the resection cavity is a dynamic process, optimal SRS/HF-SRS planning requires the timeliest MRI possible (48, 54). However, there are no guidelines either on the optimal time interval between surgery and SRS/HF-SRS. In the latest ISRS guidelines the authors did not comment on these points (32). Yuan et al. focused on the aspect of the timing of post-SRS/HF-SRS and resection and came to the conclusion that as the initial tumor size influences cavity size, and smaller metastases may profit from a longer time interval until the postoperative radiosurgery without a particular time proposal (44). Importantly, the patients mostly need a recovery period after resection that is also needed for wound healing. A time interval of at least two weeks is reasonable to our point of view and should be limited to 6 weeks postoperatively to avoid tumor recurrence. In the consensus paper by Soliman et al., 9 of 10 participants favored post-SRS within the first 4 weeks that we also favor in the routine (10). Starting radiosurgery within 30 days was also the setting in the randomized trial by Majaharan et al. (3). This is clearly one of the treatment algorithms steps that need to be standardized in the radiosurgical society in the future.

Since we focused on the NaSRS aspect, we compared the PTVs. For post-PTV we used real world data including some older cases without an additional margin, but in our recent routine clinical practice a 2.0 mm margin is now standard, as it was in the randomized phase III trial (6). The recent proposed guidelines also recommend a margin of 2.0 to 3.0 mm for the resection cavity (32). For SRS in brain metastases GTV = CTV = PTV is suggested by the German Society for Radiooncology [*Deutsche Gesselschaft für Radioonologie – DEGRO*], especially with regard to frame-based SRS treatments, with the possibility of a margin of up to 2.0 mm (55). A margin of 1.0 mm should be added in view of possible infiltrative growth of brain metastases according to Baumert et al., which is our routine practice (56). In the comparative studies for pre- and postoperative tumor volumes, only a few ones included PTVs. For instance, after a 2.0 mm margin was added to pre-GTV, the volume decrease of the originally larger tumors after resection disappeared in Atalar et al. (31). El Shafie et al. compared the PTVs of a hypothetical pre-PTV with 1.0 mm margin to different postoperative scenarios with a margin up to 3.0 mm. However, the authors did not compare the original PTV of the resection cavity treated with Cyberknife SRS with the hypothetical plans using Elekta VERSA HD linear accelerator (57). In a very recent similar comparative study by Bugarini et al. PTVs were also not significantly different between pre- and post-scenarios as in our study. In our case, based on the apparent trend, there seems to be a difference between the preoperative and standardized postoperative PTV, in the sense of greater PTV postoperatively. However, our study did not have the power to determine this conclusively. Further studies with larger case series are warranted to assess this. These authors did not present a detailed volume change dependency as in our study (33).

The amount of normal brain tissue volume receiving a relevant dose is the primary important factor regarding side effects, especially radionecrosis, but with some differences in practice for reporting that require specific guidelines to standardize data in the future. The brain volume that receives 10.0 Gy and 12.0 Gy (V10 and V12) was shown to predict the radionecrosis risk (58). For example, in SRS of brain metastases, volumes greater than or equal to 10.0 cm^3^ irradiated with 12.0 Gy (V12) were associated with a 15.0% risk of symptomatic radionecrosis (37). In the same study, three-fraction V18 < 30.0 cm³ and V23 < 7.0 cm³ were associated with less than 10.0% risk of radionecrosis in normal brain tissue (37). Another common method of assessing NBT exposure in SRS is brain minus PTV (57, 59). Zindler et al. reported for single-, three-, and five-fraction dose–volume constraints for brain minus GTV V12 = 10.0 cm^3^, V19.2 = 10.0 cm^3^, and a V20 = 20.0 cm^3^, respectively (60). Brain-GTV receiving 30 Gy was identified as a significant predictor for adverse effects in the HF-SRS series of Faruqi et al. (61) In routine clinical practice for NBT we use the following constrains regarding brain minus PTV in single session SRS V10 < 10.0 cm^3^ and for three-fraction V18 < 10.0 cm^3^ to maintain a low risk of radionecrosis (37–39). We investigated the potential benefit of NaSRS to reduce NBT exposure in a selected cohort. Because PTV margins were not standardized in this retrospective cohort, we selected cases with a 5.0 cm³ increase in post-GTV for further analysis to examine the effects of such volume increase on NBT exposure. In this preselected small cohort, we demonstrated less normal brain tissue receiving the evaluated DVH parameter for NaSRS (pre-PTV) with median 67.6% of the current standard (post-PTV), resulting in an advantage in normal-tissue preservation in NaSRS scenario. Since we kept the dosing regimen completely identical and based our hypothetical optimization on the clinical used constraints, this effect can clearly be attributed to a lower volume of the preoperative tumors. A similar advantage for normal tissue exposure was also presented in the above mentioned study favoring preoperative SRS, however, in this study the authors also changed the dose regimen for preoperative scenario (33). Because we wanted to evaluate only the volume effect on NBT exposure, we kept the SRS schedule completely identical and ensured with a robust template-based workflow in a stepwise procedure and equivalent coverage, an unbiased comparison between pre- and postoperative radiosurgery in the simulation.

We are aware of the bias within this planning study due to potential changes between pre-op and post-op conditions affecting optimal dose regimens and consecutively the planning constraints. However, as the optimization template was created for the clinical post-PTV scenario, a better set of planning parameters might have been possible within the hypothetical planning study, potentially further increasing NBT sparing. Additionally, as the volume increase was not present in all cases after resection, the different possible dose regimens in NaSRS should also be further compared to dose regimens in post-SRS. The comparability of our results with the study by Bugarini et al, in which the dosing regimen was adjusted for preoperative simulation, is very encouraging for NaSRS.

An important issue is the unintended residual tumor after surgical resection of brain metastases, which reached 15.8% in a recent study of 150 patients (53). Comparably, we observed 15.4% residual tumor in our series, however, with the caveat that we did not include only MRIs within 48 hours as this was a retrospective cohort that was not investigated by early MRI regularly. Since the dose regimens in the ongoing studies vary and are sometimes far below routinely applied doses such as 12.0 Gy, it is of great importance to select patients well for NaSRS. For *in situ* brain metastases a single dose of at least 18.0 Gy is recommended by DEGRO (55). Rosenstock et al. found that subcortical metastases located ≥ 5.0 mm from the cortex with diffuse contrast enhancement had the highest incidence of unintended subtotal resection. The proposed MRI-based assessment allows estimation of individual risk for subtotal resection and may help identify patients who are not suitable for NaSRS with regard to the risk of residual tumor (53). However, if the dose used in NaSRS was as effective as the routine doses, then the remaining tumor would not be a limitation for NaSRS. Therefore, hypofractionated NaSRS should be considered rather than dose reduction for larger tumors, which is also a topic of ongoing studies (**Supplementary Table 1**) and has already been shown to be eligible recently (2).

Although analysis of the efficacy of post-SRS was not the primary objective of this study, we examined it to demonstrate the representativeness of the cohort in comparison to other published post-SRS studies. The sole purpose of this analysis was to demonstrate the efficacy of the treatment used in this cohort to support the evaluation of tissue exposure in this setting. With only 30 patients with heterogeneous characteristics in terms of histology, location, volume, and systemic treatments, as well as nonstandard target margin, our data on this are less valuable than previously reported prospective studies. Because of the small total number of patients, we also did not perform subgroup analyses. Nevertheless, we evaluated the local progression-free survival, which was 88.0%, slightly better than the 12-month local control of 72.0% in Majahan et al. and 61.8% in the series by Brown et al. (3, 6). The distant progression-free survival in our cohort was also within the reported range of these studies (3, 6). At 12.0 months LMD control was reported 92.8% in Brown et al., which was lower in our series with an estimated LMD-free survival of 81.0% at 12.0 months (6). This may be due to inconsistent margins applied in this cohort or target delineation differences (11). In comparison, the review article by Redmond et al. reported a median leptomeningeal failure of 14.0%, with a range of up to 22.8%, comparable to our series also highlighting the need for NaSRS concepts in the future to reduce LMD risk (32). We do not elaborate on overall survival data because systemic treatment data are underreported, and we did not analyze additional extracranial metastases in this retrospective cohort.

The major limitation of our study is the small number of patients, which makes it difficult to establish a reliable threshold for GTV volume increase and to identify additional predictors. Nevertheless, this is the first matched Cyberknife SRS treated cohort with simulation of a theoretical plan to test irradiation exposure of NBT. The purpose of this study was to facilitate further studies and to simulate discussions in clinical routine as NaSRS is already mentioned in ASTRO guidelines as a possible intervention. Our study provides insights and awakens thoughts for NaSRS concepts. For further studies, we would recommend placing more emphasis on the aspect of sparing irradiation exposure of normal brain tissue and reevaluating dose regimens to achieve sufficient doses instead of single doses as low as 12.0 Gy. This is particularly crucial regarding radioresistant tumor histologies such as renal cell carcinoma.

In conclusion, the volume change of the resection cavity seems to be dependent on the preoperative lesion size. Dosimetric analysis favored NaSRS for normal brain tissue preservation in selected cases. Since the target volume directly affects the exposure of NBT, this should be considered when making treatment recommendations for NaSRS in smaller lesions. A reliable cut-off value for the preoperative lesion size to estimate volume benefit should be determined in a larger multicenter cohort. Ongoing studies will lead the way for further benefits of NaSRS independent of the volume effect.

## Ethics statement

The studies involving human participants were reviewed and approved by Charités Ethics Committee, Berlin, Germany. The patients/participants from the prospective registery provided their written informed consent to participate in this study.

## Author contributions

Conceptualization: CS and GA. Investigation, data curation: NS, BB, GK, KK, and GA. Formal analysis: AJ, NS and BB. Statistics: GA and MN. Writing—original draft preparation: CS, GA, KK, and MN. Writing—review and editing: all authors. Visualization: AK, NS, AJ. Supervision: DS, AC, DZ, PV. Project administration: GA. Revision process. All authors contributed to the article and approved the submitted version.
