# Corrigendum: What if: a retrospective reconstruction of resection cavity stereotactic radiosurgery to mimic neoadjuvant stereotactic radiosurgery

**DOI:** 10.3389/fonc.2023.1213759

**Published:** 2023-08-08

**Authors:** Gueliz Acker, Marcel Nachbar, Nina Soffried, Bohdan Bodnar, Anastasia Janas, Kiril Krantchev, Goda Kalinauskaite, Anne Kluge, David Shultz, Alfredo Conti, David Kaul, Daniel Zips, Peter Vajkoczy, Carolin Senger

**Affiliations:** ^1^ Department of Neurosurgery, Charité-Universitätsmedizin Berlin (Corporate Member of Freie Universität Berlin, Humboldt-Universität zu Berlin, and Berlin Institute of Health), Berlin, Germany; ^2^ Berlin Institute of Health at Charité - Universitätsmedizin Berlin, BIH Academy, Clinician Scientist Program, Berlin, Germany; ^3^ Department of Radiation Oncology and Radiotherapy, Charité-Universitätsmedizin Berlin (Corporate Member of Freie Universität Berlin, Humboldt-Universität zu Berlin, and Berlin Institute of Health), Berlin, Germany; ^4^ Department of Radiation Oncology, University of Toronto, Toronto, ON, Canada; ^5^ Department of Biomedical and Neuromotor Sciences, Alma Mater Studiorum - Università di Bologna, Bologna, Italy; ^6^ German Cancer Consortium (DKTK), Partner Site Berlin, German Cancer Research Center (DKFZ), Heidelberg, Germany

**Keywords:** neoadjuvant, stereotactic radiosurgery (SRS), CyberKnife®, brain metastases (BM), preoperative

In the published article, there was an error in **Figure 6** as published.

**Figure 6:**
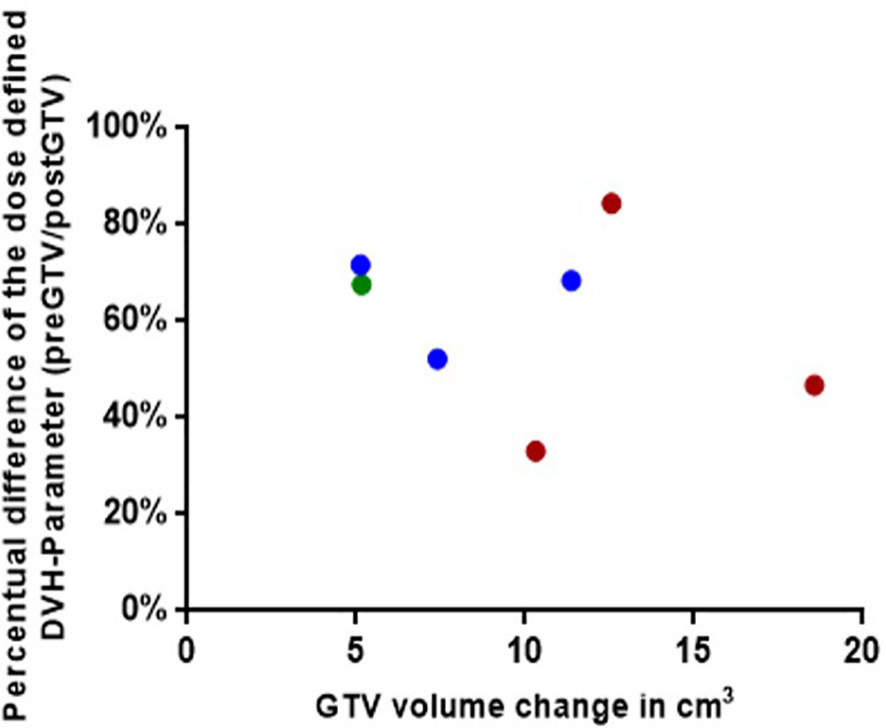
Graphical representation for the seven simulated plannings, visualizing the dose-defined dose-volume histogram (DVH) parameter ratio between simulated NaSRS and postoperative original irradiation (pre/post) against the absolute difference in GTV volume. Fractionations are color coded: Single fraction in green, 3-fractions in blue, and 5-fractions in red. GTV, gross tumor volume.

We accidentally uploaded the wrong graphic during the production process after the proofing phase as we were trying to improve the resolution of the graphic. However, the legend and caption, as well as in the text explanations of the graph are based on the correct graph and the article was also reviewed and accepted with the correct graph.

The corrected **Figure 6** and its caption appear below.

The authors apologize for this error and state that this does not change the scientific conclusions of the article in any way. The original article has been updated.

