# Supplementary material for: What if: A retrospective reconstruction of resection cavity stereotactic radiosurgery to mimic neoadjuvant stereotactic radiosurgery
Source: Front Oncol. 2023 Mar 16;13:1056330. doi: 10.3389/fonc.2023.1056330 (PMC10062706; doi:10.3389/fonc.2023.1056330)
Supplement: Supplementary file 1 [file Table_1.docx]

Supplementary Material

Supplementary Table 1*:* An overview of the ongoing registered studies

| ID | **Title** | **Registration Date** | **Study design** | **Intervention** | **Primary Endpoint** | **Secondary Endpoint** | **Expected Enrollment** | **Status** |
| --- | --- | --- | --- | --- | --- | --- | --- | --- |
| NCT00811655 | Stereotactic Radiosurgery in Treating Patients with Brain Metastases | 12/18/2008 | Interventional  Phase 2  Single group | Neoadjuvant SRS | Rate of patients with local recurrence at the Surgical Site Within 12 Months After Stereotactic Radiosurgery (SRS) | 1. Rate of salvage surgery, whole brain radiation therapy (WBRT) or SRS  2. Volume of adjacent irradiated normal brain parenchyma  3. Distal metastases  4. Quality of life after SRS assessed by FACT-Br  5. Preservation of neurocognitive functioning  6. Clinical significance of recurrent brain metastases 7. Death rate due to neurological causes  8. Overall Survival | 25 | Terminated (slow recruitment) |
| NCT01252797 | Dose Escalation/De-escalation Study of Pre-operative Stereotactic Radiosurgery for Brain Metastases (RAD 1002) | 11/22/2010 | Interventional  Phase 1 | SRS to 15 Gy in Group A (dose level II) vs. 12 Gy in Group B (dose level I) | Maximum tolerated dose (MTD) | Rate of:  - Acute treatment toxicity  - Late treatment toxicity  - Local Control  - Leptomeningeal Dissemination | 20 | Completed |
| NCT01891318 | Neoadjuvant Radiosurgery for Resectable Brain Metastases: Phase I/II Study | 6/26/2013 | Interventional  Phase 1 + 2  Single group | Neoadjuvant SRS | **Phase 1:** Determine the safety of neoadjuvant SRS at escalating doses followed by surgical resection  **Phase 2:** Determine the rate of local tumor control following treatment | 1. Rate of distant brain failure after treatment  2. Rate of salvage surgery, whole brain radiation therapy (WBRT) or SRS  3. Rate of radiation necrosis/steroid dependency  4. Radiobiologic impact of neoadjuvant SRS for resected brain metastases | 36 | Recruiting |
| NCT02514915 | Pre-operative Stereotactic Radiosurgery Followed by Resection for Brain Metastases | 7/30/2015 | Interventional  Single group | Neoadjuvant SRS | Local control rate | 1. Overall Survival  2. Distant intracranial failure 3. Health-Related Quality of Life assessed by FACT-Br | 24 | Active, not recruiting |
| NCT03368625 | Neoadjuvant Stereotactic Radiosurgery for Large Brain Metastases (NASR) | 12/4/2017 | Interventional  Phase 2  Single group | Neoadjuvant SRS | Radiation toxicity | 1. Local control  2. Leptomeningeal disease  3. Survival:  - progression-free  - overall | 30 | Recruiting |
| NCT03163368 | Dose Escalation Trial of Neoadjuvant Radiosurgery for the Treatment of Metastatic Brain Tumors | 5/19/2017 | Interventional Early Phase 1 | Neoadjuvant SRS  SRS dose determined as a function of tumor size | Maximum tolerated dose (MTD) | 1. Adverse events 2. Preliminary rates for:  - image-complete resection  - local tumor control  - intracranial control   - progression-free survival - leptomeningeal spread   - rate of salvage treatment | 25 | Recruiting |
| NCT03398694 | Pre-operative Stereotactic Radiosurgery Followed by Resection for Patients With Brain Metastases | 1/8/2018 | Interventional  Single group | Height of irradiation dose based on RTOG-9005 dosing criteria and tumor diameter  Treatment of all treatment naïve brain metastases | Rate of local control of any new, recurrent, or progressing tumors within PTV | Rate of:  - Overall survival  - Progression free survival  - New lesions outside PTV - Radiation necrosis  - Leptomeningeal spread | 50 | Active, not recruiting |
| NCT03741673 | Pre-operative SRS or Post-operative SRS in Treating Cancer Patients With Brain Metastases | 11/9/2018 | Interventional  Phase 3  Randomized  Open Label | **Experimental: neoadjuvant SRS**  SRS within 15 days of randomization followed by surgery within 15 days; additional SRS in case of recurrence  **Active Comparator: adjuvant SRS** Surgery within 15 days of randomization followed by standard of care SRS within 30 days; additional SRS in case of recurrence | Leptomeningeal disease (LMD)-free rate | 1. Local control rate  2. Distant brain control  3. Overall survival | 110 | Recruiting |
| NCT03750227 | Pre-Operative or Post-Operative Stereotactic Radiosurgery in Treating Patients with Operative Metastatic Brain Tumors | 11/20/2018 | Interventional Phase 3 Randomized | **Comparator: Post-operative SRS** Surgery followed by SRS within the following 2 weeks  **Experimental: Pre-operative SRS** SRS followed by surgery within the following 4 weeks | Central nervous system (CNS) composite endpoint event (CNS-CE event) | 1. Overall survival  2. Incidence of adverse events 3. CNS-CE event-free survival time/rate  4. Quality of Life  5. Rate of therapy completion  6. Time to:  - systemic therapy  - regional progression  - CNS progression  - Subsequent treatment  7. Rate of neurosurgical morbidity | 140 | Recruiting |
| NCT04069910 | Stereotactic Radiation Therapy Before Surgery for the Treatment of Resectable Brain Metastases | 8/21/2019 | Interventional  Randomized  Open label | **Experimental: SRS/SRT, surgery** SRS/SRT in 1, 5, or 10 fractions followed by resection within 72 hours **Active Comparator: surgery, SRS/SRT** Resection followed by SRS/SRT in 1, 5, or 10 fractions within 72 hours | Rate of leptomeningeal failure | 1. Time to progression  2. Overall survival (OS) 3. Rate of salvage treatment including surgery, SRS, SRT, or WBRT 4. Dose (Gy) and volume (cc) of radiation to adjacent normal brain parenchyma 5. Rate of distant brain failure 6. Death due to neurological causes  7. Quality of life assessment: FACTBR 8. Genetic expression profiles in pre- vs post-radiation tumor tissue | 124 | Withdrawn (no enrollment) |
| NCT04503772 | Preoperative Stereotactic Radiosurgery for Brain Metastases (STEP) | 8/4/2020 | Interventional  Phase 2  Single group | Neoadjuvant SRS  Total dose: 33 Gy á 3 fractions at isocenter, 23.1 Gy in envelope (70% isodose), i.e. 30 Gy in growth tumor volume (GTV) envelope | 6-month local control rate | 1. 1-year local control rate 2. 1-year radionecrosis rate 3. Overall survival 4. Acute (<3 months post-SRS) / delayed (>3 months post-SRS) toxicities  5. Rate of leptomeningeal relapses 6. Predictive factors for local brain control  7. Complication/prognosis factors for survival 8. Cognitive function  9. quality of life | 70 | Recruiting |
| NCT04474925 | Pre- Versus Post-operative SRS for Resectable Brain Metastases | 7/14/2020 | Interventional Phase 3  Randomized  Open Label | **Active Comparator:** Resection followed by SRS within the next 3 weeks (non-Experimental) **Experimental:** SRS followed by resection within 1 week (experimental) | Local Control | 1. Rate of:  - Distant Brain Recurrence  - Leptomeningeal Recurrence  3. Overall Survival 4. Performance on:  - Hopkins Verbal Learning Test  - Controlled Oral Word Association  - Trial-Making Tests | 88 | Recruiting |
| NCT04545814 | Analyzing Preoperative Stereotactic Radiosurgery with Gamma Knife Icon for Brain Metastases | 9/4/2020 | Interventional  Single group | SRS  Dose based to the max. tumor diameter:  - ≤ 2 cm = 20-24 Gy  - 2.1-3.0 cm = 18 Gy  - 3.1-5.0 cm = 15 Gy | 1. Subjects undergoing resection following SRS 2. Subjects with no identifiable disease following resection | Subjects with:  - CNS progression-free survival  - Overall survival  - Leptomeningeal carcinomatosis after neoadjuvant. SRS  - Radiation necrosis  - High quality of life | 10 | Recruiting |
| ChiCTR2000038995 | A clinical trial for neoadjuvant stereotactic radiosurgery before surgical resection of large brain metastasis | 10/12/2020 | Interventional  Single group | Neoadjuvant SRS | Incidence of symptomatic grade 2 radiation toxicity | 1. Local control rate of the tumor within one year  2. Incidence of leptomeningeal metastases within one year  3. Median survival time  4. Progression-free survival  5. Treatment rate of salvage therapy | 30 | Recruiting |
| NCT04895592 | Radiosurgery Before Surgery for the Treatment of Brain Metastases | 5/17/2021 | Interventional  Early Phase 1  Non-Randomized  Open Label | **Arm A: SRS, low dose dexamethasone, surgery**  SRS for 1-3 fractions over 1-5 days followed by low dose dexamethasone for 2-21 days until the day of surgical resection  **Arm B: SRS, high dose dexamethasone, surgery** SRS for 1-3 fractions over 1-5 days followed by high dose dexamethasone for 2-21 days until the day of surgical resection | Incidence of adverse events grade 3 or greater | 1. Density of immune niche in brain metastases  2. Time to local recurrence 3. Time to anywhere brain failure  4. Overall survival | 20 | Recruiting |
| ACTRN12621001329853 | Safety and Efficacy of Preoperative Stereotactic Radiosurgery for Treatment of Brain Metastases: A Phase II Study | 9/29/2021 | Interventional  Phase 2 | Neoadjuvant SRS | Symptomatic radiation toxicity (grade 2) | Rate of:  - Distant Brain Failure  - Leptomeningeal Disease  - Local Recurrence  - Overall Survival  - Post-operative outcome  - Postoperative Complications  - Progression Free Survival | 30 | Recruiting |
| NCT05124236 | Trial of Preoperative Radiosurgery Versus Postoperative Stereotactic Radiotherapy for Resectable Brain Metastases (PREOP-2) | 11/14/2021 | Interventional  Randomized  Open label | Experimental: pre-OP single-fraction SRS  Comparator: post-OP 5-Fx SRT/SRS | Incidence of Leptomeningeal Disease | 1. Local control  2. Distant brain failure  3. Radionecrosis  4./5. Quality of Life (EORTC QLQ30 and BN20) | 200 | Recruiting |
| NCT05267587 | Preop fSRS for Resectable Brain Metastases | 2/24/2022 | Interventional  Phase 2  Single group | Hypofractionated SRS (9 Gy / fraction on 3 consecutive days) followed by surgical resection within 3 consecutive days  Additional non-index brain metastasis treated with standard SRS | Time from Start of fSRS until Progression | 1. Local Control Rate  2. Rate of Leptomeningeal Disease  3. Rate of distant brain recurrence | 60 | Recruiting |
| NCT05341739 | A Phase II Study of Pre-Op SRS Followed by Surgical Resection for Brain Metastases | 4/18/2022 | Interventional  Phase 2  Single group | Neoadjuvant SRS  SRS dose preferably 27 Gy | Radiographic local control | 1. Leptomeningeal disease  2. Radionecrosis 3. Pseudoprogression | 20 | Recruiting |
| NCT05438212 | Comparing the Addition of Radiation Either Before or After Surgery for Patients With Brain Metastases Comparing the Addition of Radiation Either Before or After Surgery for Patients With Brain Metastases | 6/24/2022 | Interventional  Phase 3  Randomized  Open Label | **ARM I:** Resection followed by SRS for 1 fraction within 10-30 days **ARM II:** SRS for 1 fraction followed by resection | Time to Composite Adverse Endpoint (CAE) within 4 years after treatment | 1. Rate of:  - Overall Survival   - Local tumor progression  - Radiation necrosis  - Nodular meningeal disease   - Distant brain failures  - Frequency of adverse events 2. Change in MD Anderson Symptom Inventory - Brain Tumor (MDASI-BT) 3. Change in cognitive function | 236 | Not yet recruiting |

CNS = Central nervous system

CNS-CE = Central nervous system composite endpoint event

fSRS = fractionated Stereotactic Radiosurgery

PTV = Planned Treatment Volume

SRS = Stereotactic Radiosurgery
